# Treatment Access, Health Economics, and the Wave of a Magic Wand

**DOI:** 10.3390/curroncol29020100

**Published:** 2022-02-16

**Authors:** David J. Stewart, John-Peter Bradford, Gerald Batist

**Affiliations:** 1 Department of Medicine, Faculty of Medicine, The Ottawa Hospital, University of Ottawa, 501 Smyth Rd., Ottawa, ON K1H 8L6, Canada; 2 Life Saving Therapies Network, 173 Heath St., Ottawa, ON K1H 5E6, Canada; jp@lifesavingtherapies.com; 3 Segal Cancer Centre, Jewish General Hospital, McGill University, Montreal, QC H3T 1E2, Canada; gerald.batist@mcgill.ca

**Keywords:** drug access, CADTH, PMPRB, drug costs

## Abstract

New drugs are expensive, in part due to excessive drug development costs. Governments are trying to reduce drug prices. This can delay access to effective agents. A country’s access to new drugs correlates with prices they agree to pay. After Health Canada approves a drug, the Canadian Agency for Drug and Technologies in Health (CADTH) assesses it. CADTH’s approval is usually contingent on it costing ≤CAD 50,000 per quality adjusted life year (QALY) gained. This value (unchanged from the 1970s) is inappropriately low. An inflation-adjusted CAD 50,000 1975 QALY should translate into a CAD 250,000 2021 QALY. CADTH’s target also does not consider that drug development costs have risen much faster than inflation or that new precision therapies may only be used in small populations. In a separate process, proposals from the Patented Medicines Price Review Board (PMPRB) would decrease initial Canadian drug prices by 20%, but prices would fall further as sales increased, with ultimate price reductions of up to 80%. PMPRB claims its proposal would not reduce drug access, but multiple analyses strongly suggest otherwise. Government price controls target the symptom (high prices), not the disease. They translate into shortages without solving the problem. CADTH and PMPRB approaches both threaten access to effective drugs.

## 1. Introduction

Most metastatic cancers remain incurable. Despite this, a rapidly emerging spectrum of new anticancer drugs has enabled many patients with advanced cancers to enjoy symptomatic improvement and longer lives.

However, various factors delay the access of Canadian patients to these effective new therapies. The most important factor that causes delays is the time required for clinical trials that are needed to demonstrate drug efficacy. Additionally, contributing to the delays are the time required for Health Canada review and approval of the drug and the time required to determine whether and how the drug will be funded.

## 2. Drug Development and Clinical Trials

It takes too long to accrue the data required for drug approval. This delay has increased steadily. In the 1960s, it took an average of eight years to take a drug from discovery to approval. Now it takes 12–15 years [1]. The major contributing factor is the time that it takes to set up and conduct clinical trials [2,3]. Most drugs will require a positive randomized clinical trial (RCT) for approval, and it can take a long time to complete this.

As reported elsewhere, several factors slow clinical trials and thereby contribute to the long timelines for drug development [4,5]. For many of these factors, there is little evidence that they contribute much value.

Some people have even advocated regulatory changes that would further slow the development and availability of new anticancer drugs. Their rationale is that these changes would lessen the potential negative impact of cancer spending on other areas of healthcare [6]. To us, this is a very misguided “crabs in a barrel” approach. It ignores the fact that cancer is by far Canada’s highest cause of death [7] and of life-years lost [8]. It also tacitly promotes a system that condones increased suffering among terminally ill patients.

Worldwide, tens of thousands of life-years may be lost for each year of delay in the approval of an effective new therapy. We urgently need major reforms that are lifesaving by being timesaving [9].

## 3. Expedited Access

Fortunately, if early clinical experience with a new drug indicates that it is highly effective, it may be made available to patients without the prerequisite of further trials. It may be approved rapidly in the United States through the FDA’s “breakthrough therapy” designation [10] or in Canada by Health Canada’s “Notice of Compliance with Conditions” designation [11]. This expedited approval may be conditional on further trials being performed subsequently.

Rapid access is particularly important for effective new agents that are the first in their class and that provide benefit to patients with lethal diseases such as metastatic cancers. In this situation, there may be no reasonable alternatives. If a new agent is a “me-too” drug that works in the same way as a currently available agent and does not have any obvious advantages, rapid access may be a less pressing issue.

With accelerated access through approaches such as “breakthrough therapy” designation, there is always some risk that subsequent trials may fail to confirm that the therapy offers sufficient benefit to justify ongoing access. If this happens, the therapy may be withdrawn from the market after discussion between regulators and the pharmaceutical company, or regulators may unilaterally rescind approval [12].

In other cases, a therapy may remain approved despite failure to meet its primary endpoint in subsequent trials [12]. This can happen if the drug has other benefits that are thought to be useful. For example, in many tumor types, immune checkpoint inhibitors appear to be of marked benefit in one patient subpopulation and of little benefit in another [13]. Consequently, a comparison to other therapies may fail to reveal a survival benefit in the overall population, but ongoing access might be considered important due to the unequivocal benefit derived by a substantial subpopulation of the patients.

## 4. Drug Approval Processes

Once sufficient clinical trials data are available to support an application for drug approval, many companies apply first to the United States (US) Food and Drug Administration (FDA). They apply to the US first because there is a high potential for profit in the American market. With 330 million people, the US is a large market. On average, Americans pay far higher prices for medications than citizens of other countries [14]. High American prices are due in part to the Medicare Prescription Drug, Improvement and Modernization Act of 2003 that stipulates that US Medicare is not permitted to negotiate prices. Instead, US Medicare generally must accept the price named by the pharmaceutical company [15].

Companies next typically apply for approval to the European Medicines Agency (EMA). The EMA reviews drugs on behalf of 27 European nations, which have a combined population of more than 500 million people.

Price negotiations follow approval. The time this takes varies from country to country. Some European countries take two years or more to make a deal with the company. On the other hand, Germany requires an average of only 4.6 months to conclude price negotiations [16]. Germany typically pays prices that are lower than American prices, but higher than many other countries [17].

The FDA charges companies approximately USD 2.9 million for an application for potential access to 330 million Americans [18]. The EMA charges approximately EUR 300 thousand for an application that would permit access to 500 million Europeans [19]. Meanwhile, Health Canada charges roughly the same amount as the EMA (CAD 437 thousand) for potential access to just 38 million Canadians [20].

Completing the applications for each jurisdiction is labor intensive. The process is different from one jurisdiction to another. The high cost of applying to the relatively small Canadian market is an important reason for delays. Applications to Canada follow those to the US by an average of ten months and those to the EMA by an average of eight months [21].

However, Canadians generally pay high prices for their drugs [22]. Canadian drug prices are far lower than American prices, but they are similar to the highest European prices [17]. Paying high prices means that Canadians gain access faster than many other countries, despite the relatively small size of the Canadian market.

**Figure 1:**
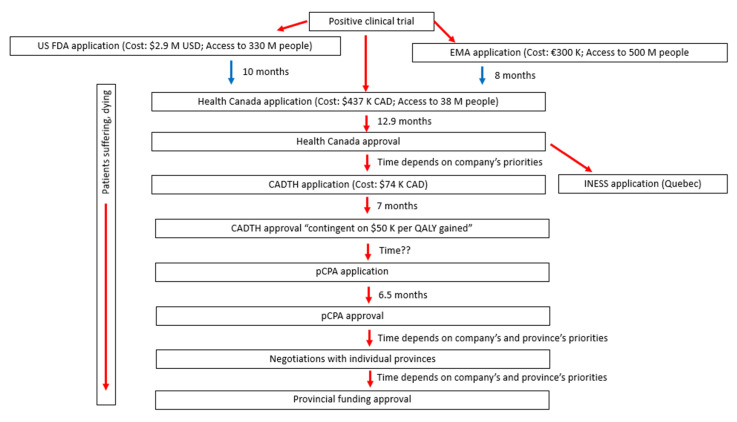
Time for drug approval and funding in Canada. US FDA: United States Food and Drug Administration; EMA: European Medicines Agency; CADTH: Canadian Agency for Drug and Technologies in Health; INESS: Institut National d’Excellence en Sante et en Services Sociaux; pCPA: pan-Canadian Pharmaceutical Alliance.

Health Canada typically takes slightly longer than the FDA or EMA to review an application [23]. All things considered, Canadians access new agents an average of 1.3 years after Americans and Europeans [24]. Once Health Canada grants approval, Canadians may purchase the therapy if they pay out of pocket or if they have private insurance. Timelines for approval by Health Canada are outlined in Figure 1.

Project Orbis involves the simultaneous assessment of a new therapy by different countries at the same time. It was launched by the US FDA, Health Canada and Australia in 2019 [25]. Other countries have been added since. Project Orbis potentially could speed up Canadian access to new drugs. The project is still in its early days. It is too soon to assess how much of a difference it will make.

## 5. Drug Funding

In his 1976 Karnofsky Lecture, the late Emil J Freireich presented Freireich’s Laws [26]. One of his seven laws was “Always be prepared for success!”. Sadly, we are not prepared. The cost of individual therapies is rising rapidly, even as more effective new cancer medications become available [27].

It can be challenging to decide on a reasonable price for a new drug if the agent is highly innovative and meets an unfulfilled need, but the company is asking a very high price. Various new approaches to evaluate a drug for funding have been proposed [28], but there is no perfect method to determine what should and should not be funded.

In Canada, the current approach to funding drugs involves a slow and complex bureaucratic labyrinth, as illustrated in Figure 1. Health Canada’s approval of the drug is only the first step. After that, a company pays CAD 74,030 to submit an application for a Health Technology Assessment (HTA). This HTA is a prerequisite for public funding of the drug through provincial healthcare systems [29].

First, the HTA application goes to the Canadian Agency for Drug and Technologies in Health (CADTH) to assess the “need” for the drug and the drug’s efficacy and cost-effectiveness. CADTH takes an average of seven months to complete their review of a drug that Health Canada has already approved [30]. CADTH reviews drugs for all provinces and territories except Quebec. Quebec has a separate review process through its Institut National d’Excellence en Sante et en Services Sociaux (INESS) [31].

If approved by CADTH, the drug then passes to the pan-Canadian Pharmaceutical Alliance (pCPA), which negotiates a price with the company on behalf of all provinces and territories except Quebec. pCPA aims to complete its negotiations in 6.5 months [32].

Individual provinces may then conduct their own additional negotiations, which can prolong the delay.

After negotiations between a drug company, the pCPA and the provinces are completed successfully, the price is kept secret. However, the Ontario Auditor General’s annual report suggests that the price is about 30% less than the official “list price” for the drug in Canada [33]. Keeping the price secret means that a company can offer the drug at a lower price than if the price is public. If a low Canadian price is made public, this could be used by other countries to also try to lower their prices. Keeping this price secret probably benefits Canadians since it means their provincial governments generally will pay a lower price than if the price is public.

## 6. Impact of Cumulative Delays

Clearly, we are entangled in a multi-staged, inefficient muddle. The extensive impediments [4,5,34] to rapid execution of clinical trials can mean huge numbers of life-years may be lost worldwide before there are sufficient data to apply for drug approval [9].

Thousands of additional Canadian life-years may be lost due to delays in drug companies applying for Canadian approval plus the year or more that it takes to fund a new drug after it is approved by Health Canada [35].

## 7. The Health Economist’s Magic Wand

There are several troubling aspects of Canadian price negotiation processes. For example, CADTH has embraced some metrics apparently conjured up by a health economist’s magic wand. Specifically, CADTH has typically made its approval of a new agent conditional on its target price being no higher than CAD 50,000 per quality adjusted life-year (QALY) gained by the new therapy. For some therapies, this would require a price reduction of 80% or more [36].

Everyone likes a deal, so this may sound very attractive. However, this type of “value-based pricing” has several serious pitfalls [37]. The first is that the target of USD 50,000 per QALY gained was first proposed in the 1970s [38]. Adjusting for inflation, a 2021 dollar is worth less than 20% of a 1975 dollar [39]. Therefore, if USD 50,000 was the value of a QALY gained in 1975, then it should be USD 250,000 or more in 2021. Effectively, CADTH’s 2021 price target of CAD 50,000 per QALY gained means that the wave of a health economist’s magic wand has erased more than 80% of the inflation-adjusted value of a Canadian life compared to five decades ago.

The decrease in value is even greater than 80% because the 1970s target was in US dollars and CADTH’s current target is in cheaper Canadian dollars. What economic alchemy justifies this?

We find that this arbitrary devaluation of a Canadian life by CADTH makes it difficult to take CADTH seriously.

## 8. The Abracadabra of QALY Calculation

Typically, QALYs gained are calculated based on differences in outcome between the new therapy and older therapies in a randomized clinical trial (RCT). However, there are several ways in which RCTs can mislead [40]. Furthermore, the quality of life (QOL) assessments that are needed for the calculation of a QALY are themselves very imprecise metrics with several intrinsic flaws and biases [41]. Given these, regulators have rarely approved a new therapy based predominantly on QOL assessments. Our experience suggests that clinicians tacitly agree. They rarely base therapy decisions on QOL assessments [42]. Instead, they generally use response rates, survival and toxicity data to judge the value of a drug.

Therefore, how reliable is CADTH’s calculation of QALYs gained? Why do government health economists advocate the use of this metric? We would expect that the 95% confidence intervals for the number of QALYs gained in many cases would be extremely wide if all relevant imprecisions, flaws and biases were taken into consideration. We do not recall any CADTH funding recommendations that acknowledge the potential impact of wide QALY confidence intervals.

Crossover in clinical trials can introduce further substantial uncertainty into the calculation of the number of QALYs gained [43,44]. The control arm will do better than it should if a new drug is effective and patients on the control arm are permitted to cross over when their disease progresses [45]. Consequently, the QALYs apparently gained with the new therapy will be less than the number actually gained. How does the health economist’s magic wand calculate crossover-adjusted QALYs gained for a new drug? Are their methods evidence-based?

In general, the more effective a drug, the more likely that crossover will be permitted in studies assessing it [45]. Hence, the most effective agents are likely to be hurt the most.

Some trials have tried to avoid this issue by forbidding crossover. The possibility of having access to an effective new agent may be sufficient to coerce patients into participating in a trial despite crossover being forbidden. However, we and others [46] find it unethical to forbid crossover.

Breakthrough drugs are approved because they are judged to be highly effective based on a high response rate in a single arm non-randomized trial [47]. How is one to determine the QALYs gained with a new breakthrough drug? Again, the more effective the agent, the more likely that it will be approved without an RCT and the more problematic any calculation of the QALYs gained.

There is yet another problem. The cost to bring an effective new drug to market has increased much faster than inflation over the past several decades, despite CADTH’s view that the value of a Canadian life is inflation-proof. In 1962, it cost about USD 4 million to bring a drug to market. By 1987, this had increased to USD 231 million, and by 2013, the average cost was USD 2.9 billion [3,48]. The primary driver for this increase was the very high cost of the clinical trials required for drug approval [3]. These clinical trials costs, in turn, are driven largely by regulation that is essential but that is not cost-effective [4,37].

Approval for every individual drug does not cost USD 2.9 billion. Instead, USD 2.9 billion is the average cost per drug if the amount spent on research by a pharmaceutical company is divided by the number of new drugs approved [3].

Only 3–8% of cancer drugs that enter clinical trials are eventually approved [49,50,51]. Profits from the successes must pay for not only the cost of development of the successful drugs, but also the high costs of the failures. No company would invest in new drug development if it had only a 3–8% chance of success and was not permitted to recoup its huge losses on the failures [37]. New drug development and progress against cancer will halt unless the costs of failures can be recouped from sales of the successes.

Therapy is becoming increasingly more personalized. For example, in the case of advanced non-small cell lung cancer (NSCLC), a drug that targets a specific mutation may be used only in the small fraction of patients that have that mutation.

Therapy personalization is a mixed blessing. The number of patients who are candidates for a therapy decreases substantially as therapies become more personalized [34,52]. On the other hand, the probability of a benefit from the agent is increased for those who are candidates for the therapy [34].

It may cost the same amount to bring a drug to market if the target population is small or large, unless the drug is approved as a breakthrough therapy, without the requirement for an initial or confirmatory RCT. Hence, to recover drug development costs, much more must be charged for a drug relevant to a small population than for a drug targeting a large population. If these costs cannot be recouped, then no company will waste time trying to develop a drug for a small population. We might wind up with many great new drugs to treat common conditions such as runny noses and constipation, but a patient with an uncommon cancer will be out of luck.

However, CADTH has banished this reality into oblivion with another wave of the health economist’s magic wand. We ask again, can Canadians really take CADTH seriously?

## 9. The Patented Medicines Price Review Board (PMPRB) Complicates Matters

In a Kafkaesque twist, the Canadian government has two separate bodies weighing in on drug pricing. They are CADTH with its CAD 50,000 per QALY-gained dictum and the PMPRB.

PMPRB sets the maximum prices companies can charge. They have proposed new regulations that would further decrease the price a company could charge when they first launch a drug. If enacted, these new rules would also force companies to reduce their prices as sales of a drug rise [53,54]. These regulations were initially scheduled to come into effect on 1 January 2022, but this has just been pushed back to 1 July 2022. We would like to see them shelved completely. We are not alone.

CADTH and PMPRB each represent the Canadian government, yet they set different prices and conditions for pharmaceutical companies. Kafka would be proud. PMPRB’s proposed new regulations set the initial value of a QALY gained at up to four times higher than the CADTH QALY value, and this is good. However, in PMPRB-land, the QALY value would drop sharply as sales of the drug increase [53,54]. We have simplified the situation somewhat, but PMPRB rules are quite complex. By some estimates, Canadian prices for some new patented agents would drop by 50–80% under the proposed new PMPRB rules [55].

As outlined in a separate commentary in this issue of *Current Oncology*, many physicians (and patient groups) are concerned by these new PMPRB regulations. Published evidence indicates that companies may delay application for approval for their agent(s) in countries with low drug prices [56,57,58]. In keeping with this, several companies have stated that the proposed PMPRB regulations will result in either delaying or completely foregoing an application for Canadian market approval [59].

In presentations to the Canadian public, PMPRB has argued that price controls are necessary to contain rising drug expenditures [53]. On slide 34 of their presentation, they pointed out that health care spending in Canada is rising as a percent of GDP. To support their claim that tighter controls on patented medicine prices are needed, they also stated that “Patented drugs account for an increasing share of health care spending: 7.5% in 2017, up from 6.3% in 2013” [53]. However, there is a catch. According to the PMPRB’s own published data, spending on patented medications as a percent of GDP remained relatively constant from about 2002 to 2019 [17]. There were a few minor aberrations over that time period. From Figure 3c of their publication [17], patented medicine sales as a percent of GDP peaked at 0.79% in 2004 and was 0.75% in 2019. The lowest it went from 2004 to 2019 was 0.71% in 2013, and the highest it went was back up to 0.78% in 2017. Spending in 2013 was particularly low and spending in 2017 was particularly high. In their public presentation [53], PMPRB displayed the difference between 2013 and 2017 as an indication of skyrocketing expenditure on patented medications. They failed to disclose the fact that these two years were both outliers! We wonder whether they were deliberately trying to dupe a naïve Canadian public. Or perhaps they simply do not recognize the problem with their analysis.

Either way, we see this as a problem. We would be happy to hear from them if they have another explanation.

We also feel that slide 43 of the PMPRB public presentation [53] is misleading. PMPRB appears to want the public to believe that cutting drug prices would not result in reduced or delayed Canadian access to effective new drugs. The slide includes the statement “Countries with lower patented drug prices than Canada may have greater availability of new medicines”. However, they present data from a spectrum of European countries as if they are independent events, with some European countries with low prices gaining relatively rapid access.

An important reason that these countries gained relatively rapid access is that they are part of EMA. They thereby benefitted from the companies trying to access the rich markets in Germany and similar countries. Other European countries were dragged along despite low prices. Independent calculations indicate that price does indeed have an important impact on access [58]. The bottom line is that while a few countries with low prices “may” have good access, the majority do not.

To assess this further, we used data presented by the PMPRB [53] to rank European countries according to average drug prices paid. We then extracted data from a publication on European access to drugs and ranked European countries according to how rapidly they got access to new drugs [16]. These two metrics correlated very strongly, with a Spearman coefficient of 0.65 (*p* = 0.018). This analysis indicates that starting from the common timepoint of EMA approval, a country’s subsequent access to a drug is highly correlated with the amount the country is willing to pay for the drug.

We also used data from PMPRB’s presentation [53] to correlate the percent of new drugs available in a country with the average price a country pays, across the range of 31 European and non-European countries included in the PMPRB presentation. The price paid correlated significantly with the percent of new drugs available in a country (Spearman r = 0.45, *p* = 0.01). For European countries, the Spearman coefficient was 0.70 (*p* = 0.0002).

Again, due to their EMA membership, a few European countries that paid lower prices than Canada had equal or better access compared to Canada. However, none of the five non-EMA countries paying less than Canada has as good a drug access as Canada, and 65% of all countries paying less than Canada have poorer drug access.

PMPRB proposes that initial maximum Canadian prices for drugs introduced in the future would be lower than they are currently. Specifically, Canadian prices for new drugs would be set at the average price charged by a new comparator group of 11 countries (Australia, Belgium, France, Germany, Italy, Japan, Netherlands, Norway, South Korea, Spain, Sweden, and the United Kingdom) [60]. This grouping is designed to bring the initial price Canada pays for medications down by 20% or more to the median for the Organization for Economic Co-operation and Development (OECD) [60]. As noted earlier, the proposed rules that would come into effect as sales of the drug rise could result in Canadian drug prices falling overall by 50–80% [55]. The assessment of data from PMPRB’s public presentation [53] clearly indicates that, on average, the number of new drugs available to most countries paying these reduced prices would be substantially lower than the numbers of drugs to which Canadians currently have access [53].

Not only might new drugs not be launched in Canada, but PMPRB’s requirement of a price reduction as sales rise [55] could have further negative consequences. History tells us that if a country cuts the price of a drug that has already been launched, a company may then withdraw the drug from that country [57]. Why would PMPRB try to make us believe that companies would treat Canada differently than they have previously treated some other countries?

In their presentation, was PMPRB trying to deliberately mislead an uninformed Canadian public about the risks associated with their proposal, or—equally disturbing—were they simply blithely unaware of these risks, despite many people pointing them out to them? Alternatively, perhaps—like CADTH—they too have a magic wand to make the problem go away. If they have another credible explanation, we would be happy to hear from them.

## 10. Potential PMPRB Impact on Canadian Healthcare Research

The PMPRB changes may also mean that companies will be less likely to conduct clinical trials in Canada or make drugs available to Canadians on a compassionate access basis [59]. This is unacceptable.

Companies are generally more likely to conduct clinical trials in a country if they plan to eventually launch their new drug in that country. Conducting a clinical trial in the country is a mechanism by which a company can familiarize clinicians with the therapy. In addition to companies stating that they would be less likely to conduct clinical research in Canada if new PMPRB rules came into effect [59], some authors feel that there is already evidence that this has happened, in anticipation of the PMPRB rules [61].

Any major reduction in clinical research activity in Canada will need to be monitored carefully, as this could have major negative consequences. Participation in clinical trials is a way that patients may access effective new therapies. For Canadian patients, this opportunity could be reduced if clinical research activity decreases.

Additionally, of concern is the fact that many healthcare research initiatives in Canada require funding from federal and provincial governments, with matching funding from pharmaceutical companies. The contribution from the companies often constitutes 80% or more of the total funding. In this way, a healthy, collaborative relationship between government and industry fosters Canadian healthcare research, while a reduction in industry commitment to investment in Canada risks threatening it. This funding is essential to the success of Canadian healthcare researchers, and Canadians derive substantial direct and indirect benefits from these Canadian research activities. It will be to Canada’s detriment if this collaboration is lost.

## 11. Should PMPRB Be Dissolved?

Serious concerns have been raised about various other PMPRB actions as well. Some prominent voices have called for PMPRB to be dissolved [62]. We strongly endorse this. Given the roles of Health Canada and CADTH, PMPRB is at best redundant. At worst, it threatens both Canadian healthcare research and the access of Canadians to effective new agents.

## 12. Government Price Controls Do Not Work

Both CADTH and PMPRB appear to think that government price controls will bring down the high cost of medications. Some government health economists believe their magic wands will achieve this, but history and mainstream economic evaluations tell us that this will not work. Government price controls ultimately create shortages [63]. What would a Canadian shortage look like? Simply put, several effective new drugs would not be available here. Or they would be greatly delayed. Either could translate into suffering and loss of life that could have been avoided with the new drugs.

Attempts at government price controls also often result in end runs and “special considerations” for politically powerful interest groups. These ultimately result in higher prices rather than lower prices [63]. The COVID-19 vaccine may be a case in point. Initially, there was no plan for COVID-19 vaccines to be exempted from the planned new PMPRB rules. However, an exemption for these vaccines was negotiated, and the vaccines rapidly became available to Canadians. Cole Pinnow from Pfizer Canada indicated that Pfizer might not have moved forward with their COVID-19 vaccine as rapidly as they did in Canada without this exemption. They had lots of other countries eager to buy it if Canada did not want it [64]. Was this an empty threat by Pfizer or was it real? We may never know, but we do know that an exemption was granted, and that such exemptions are typical of the end runs and special considerations that so frequently castrate government attempts at price controls [63]. The Canadian public has no idea how much these vaccines are costing us.

## 13. High Drug Prices

High drug prices are a huge problem. However, CADTH and PMPRB solutions simply will not work. Government wage and price controls did not work as a tool against inflation in the 1970s since they attacked the symptom (inflation) rather than the underlying cause (governments printing too much money) [63]. Mainstream economic theory tells us that you must tackle the cause if you want to beat a problem. It does not help to simply suppress the symptom.

Several factors contribute to high drug prices [37]. The incredibly high cost of new drug development is a major one. If we try to suppress drug prices without tackling the high cost of new drug development, the long-term consequence will be that we will halt investment in new drug development.

There are many ways to cut the costs of new drug development markedly, while speeding up access to effective new therapies that can alleviate suffering and prolong lives [4,5,34,37]. For example, the extremely high cost of excessive documentation requirements in clinical trials is an important factor driving high drug development costs. These high documentation costs have been exacerbated substantially by the rapidly increasing involvement of Clinical Research Organizations (CROs). This would be a good place to start in addressing the high drug development costs that ultimately translate into high drug prices.

However, this is only the beginning. We need to look at all the inefficiencies and impediments that unnecessarily drive up drug development costs while slowing access to effective new drugs [4,5,34,37]. We need a progress-centered framework for clinical research in lethal diseases [5]. Instead, the Canadian government wants its health economists to wave their magic wands with ineffective price controls. We have been asking governments for years to take effective action to cut drug development costs while speeding progress, but they largely have failed. Who is in charge here anyway?

## 14. Diagnostics, Staging and Therapy Delivery

There is an exaggerated focus on drug costs while other aspects of the healthcare system lag. To derive the full potential benefit from the effective new tools as they become available, we need to pay attention to all required components of the healthcare system.

Potent new drugs that governments fund can produce cost-effective outcomes, with reduced suffering, improved quality of life, and longer survival. However, patients need to actually receive these drugs for this to happen. These therapies cannot be started until a patient has completed the required diagnostic and staging tests to confirm that they are a candidate for the therapy.

There are several impediments to the rapid execution of these tests. The testing often requires an assessment by a specialist to perform the required diagnostic procedures. Scans are then generally required to determine the cancer stage. However, Canada is tied for the worst access to specialist care among twenty evaluable OECD countries [65]. Once a Canadian sees a specialist, it then typically takes weeks to organize the necessary tests and procedures [66].

Once a biopsy is finally set up, the specimen must be assessed by a pathologist. In Canada, the number of pathologists per million population dropped by 43% between 1995 and 2019 [67].

Scans are generally required to determine the cancer stage. With respect to access to these scans, Canada ranked 30th out of 36 evaluable OECD countries for CT scanners in 2019 [68], 27th out of 35 for MRI scanners [69], and 20th out of 30 for PET scanners [70]. (All figures are in scanners per million population.) Canada has fewer CT scanners per million population than Turkey, Chile, Greece and many Eastern European countries [68].

Canada has fewer medical oncologists than nephrologists, gastroenterologists, pulmonologists, neurologists, infectious disease physicians, cardiologists or general internists (and markedly fewer medical oncologists than psychiatrists) [71]. Despite this, the wait times for Canadian patients to see medical oncologists is shorter than for almost any other non-emergency specialty [72].

However, it then often takes too long to perform the required testing before therapy can be initiated.

Some cancers are relatively indolent. However, others progress rapidly and require therapy to start promptly. For some common cancers, death rates rise by about 1–3% for every week that therapy is delayed for patients with early-stage disease [73]. In advanced NSCLC, 4% of patients die every week that therapy is delayed [74]. Others deteriorate to the point that therapy initiation is no longer feasible.

All of us who treat lung cancer have seen this happen. This is a key reason that only 25% of Ontario patients with advanced NSCLC ever make it onto treatment [75].

It is not good enough that governments pay for effective new drugs. They also need to do a better job of providing better and faster access to the necessary diagnostic and staging tests that are needed before these new therapies can help a patient.

Older therapies and approaches may be cheaper than newer ones. However, they also can be much less effective. This can translate into increased clinic and emergency room visits, hospitalizations, and the use of other aspects of the healthcare system. It can also mean increased suffering and premature deaths.

At play is what we have called “the Glieberman Principle”. When real estate developer Bernard Glieberman bought the struggling Ottawa Rough Riders in 1991, the Ottawa Citizen reported that he had told his general manager to “Go out and hire the best coach that money can buy. The second best is too expensive!”.

The same can apply to healthcare. Doing things the old, second best way can translate into increased costs that can be avoided by using new, better approaches. This is why we now use new anticoagulant approaches instead of admitting patients with clots for a week of intravenous heparin. It is why we use bisphosphonates for hypercalcemia instead of admitting patients for a week of hydration. It is why we use Pleurx catheters for pleural effusions instead of admitting patients for a chest tube. It is why we perform PET scans to look for metastatic disease instead of putting patients through surgery or radiotherapy that is destined to fail. A long list of additional advances could be added to this.

Unequivocally, we must pay attention to bringing down costs, but there is a thin line between saving money by avoiding expensive new approaches and increasing costs by using old, ineffective approaches. We need better data infrastructures to enable us to more effectively assess this.

Compounding the delays in drug access and the shortages of resources, unspoken forces coerce some physicians into remaining silent. Both the Canadian Medical Protective Association and the College of Physicians and Surgeons of Ontario mandate that if a physician feels a patient needs something, then they must deliver it. Failure of the government to provide the required resources is no excuse. Faced with an impossible situation, many physicians remain silent rather than taking the risk of speaking out.

During the 2021 election campaign, Prime Minister Trudeau told the country that he would hire hundreds of additional physicians for Canada. However, where will he find them? It takes years to train one. From what poor country does he plan to acquire them? Or, will that also only require another wave of a magic wand?

## 15. Cancer Outcomes

Canadian cancer patients’ survival is consistently lower than that of Americans [76,77]. In particular, cancer survival in Canadians is lower than in Caucasian Americans but similar to the underserved African American population [77]. The poorest Canadians in the lowest income quintile have a higher probability of surviving cancer than the poorest Americans, but Americans in the top four income quintiles have a higher probability of surviving cancer than their Canadian counterparts [78].

Canadian life expectancy was 7th longest in the world between 1985 and 1995 [79]. In 2020, it was 16th [80]. Life expectancy in Canada is projected to drop to 27th in the world by 2040 [81]. We are concerned that impaired access to effective new agents and to required diagnostic and staging resources will accelerate this trend.

Come on, Canada. It is time to wake up. We can do better. We cannot rely on magic wands to fix this, no matter how much faith the Canadian government and its health economists may have in them. With the development of vaccines for the current pandemic, the Canadian population has seen what can be accomplished if we attach a sufficient sense of urgency to it. Can we accept that the saving of lives of Canadians with cancer is any less urgent than COVID-19?

## Data Availability

Not applicable.
